# Comment on “Large Bottleneck Size in Cauliflower Mosaic Virus Populations during Host Plant Colonization” by Monsion et al. (2008)

**DOI:** 10.1371/journal.ppat.1005512

**Published:** 2016-04-14

**Authors:** Gaël Thébaud, Yannis Michalakis

**Affiliations:** 1 INRA, UMR 385 BGPI, Montpellier, France; 2 MIVEGEC, UMR 5290 CNRS IRD Université de Montpellier, Montpellier, France; University of California San Francisco, UNITED STATES

Our research groups have previously published a study estimating the bottleneck size of *Cauliflower mosaic virus* (CaMV) during host plant colonization [1]. Two methods were used in that study, both based on the temporal evolution of neutral marker frequencies; one uses estimates of *F*_ST_, and the other tracks changes in marker frequency variance over time. Here we report that (i) the variance-based method was actually published by Felsenstein [2], a reference we were not aware of when publishing the original study; (ii) equation (4) in [1] is actually an approximation; and (iii) these methods rely on the assumption that the bottleneck size is constant, which can be relaxed by assuming it is a Poisson-distributed random variable.

Equation (4) in [1] reads *N*_*v*_ = *p*(1−*p*)/[Var(*p*’)−Var(*p*)], where *N*_*v*_ is the estimated bottleneck size, and *p* and *p*’ represent the frequency of a neutral marker before and after the bottleneck, respectively. The approximation in this equation concerns the numerator. Its exact formulation should be *E*(*p*(1 − *p*)) and not *E*(*p*)(1 − *E*(*p*)), which is the expression implied in [1] and used in the numerical application on CaMV (*E* denotes the expected value). Based on the definition of the variance, it can be shown that *E*(*p*(1 − *p*)) = *E*(*p*)(1 − *E*(*p*)) − *Var*(*p*), which is equivalent to the numerator of equation (14) in [2]. Thus, the approximation will give relatively accurate results whenever *Var*(*p*) is negligible relative to *E*(*p*)(1 − *E*(*p*)). This was indeed the case in the study by Monsion et al. [1], as can be seen in Table 1, which compares the *N*_*v*_ values published in [1] to those obtained when applying the exact expression. The results of that paper are thus not qualitatively affected and, consequently, its conclusions remain unchanged. This method was also used in another study [3], the results of which are also marginally affected quantitatively, but not at all qualitatively (Table 2). To illustrate the application of these methods in a situation in which the estimated bottleneck size is lower, we used data from Tromas et al. (Table 3) [4].

**Table 1:** Comparison of estimates of *N*_*v*_ using the approximate formula published in [1] (values in Table 2 of this publication), those using the exact expression, and those assuming that bottleneck size is a zero-truncated-Poisson-distributed random variable.

Marker	*N*_*v*_ approximate	*N*_*v*_ exact	*n*_*vP*_
VIT1	423.00	421.16	422.16
VIT3	298.45	296.31	297.31
VIT4	484.61	481.42	482.42

**Table 2:** Comparison of estimates of *N*_*v*_ published in [3] using the approximate formula (values in table S1 of [3]), those using the exact expression, and those assuming that bottleneck size is a zero-truncated-Poisson-distributed random variable. Note that due to copying errors, the values reported in table S1 of [3] for leaves 5 and 21 were slightly wrong—we report here the correct values.

Leaf level	*N*_*v*_ approximate	*N*_*v*_ exact	*n*_*vP*_
**# 5**	8.78	8.63	9.79
**# 16**	127.70	116.98	117.99
**# 21**	15.45	13.90	14.99

**Table 3:** Estimates of *N*_*v*_ using data published in [4] for leaf level 5. The authors of that paper had estimated a bottleneck size of 6 (see their figure 5B) using the *F*_ST_ method. To obtain the reported estimates, Leaf 3 and Leaf 5 were considered as the levels before and after the bottleneck, respectively. From the histogram 4A in [4], we calculated a mean frequency of 0.622 for the marker in Leaf 3, and we used the variances in marker frequency reported for Leaf 3 and Leaf 5.

Leaf level	*N*_*v*_ approximate	*N*_*v*_ exact	*n*_*vP*_
**# 5**	6.91	6.83	8.02

Both Felsenstein’s formula [2] and its approximation assume that the bottleneck size *N* is constant (i.e., identical in all the sampled plants). However, we can make the more realistic assumption that *N* follows a zero-truncated Poisson (ZTP) distribution (i.e., *N* can vary among experimental replicates according to a Poisson distribution, but *p*’ cannot be measured when *N* = 0 and the plant is discarded, hence the zero-truncation):

$$N∼\text{ZTP}\left(n_{vP}\right),\;\text{so}\;\forall k\in N*,P(N=k)=\frac{n_{vP}^{k}}{k!(e^{n_{vP}}-1)}.$$

Among the *N* genomes that go through the bottleneck, the number *X* bearing the neutral marker is drawn according to its pre-bottleneck frequency *p* from the binomial distribution: *X*~B(*N*,*p*). Thus, the marker frequency after the bottleneck is *p*’ = *X*/*N*. The variance of this marker frequency can be expressed as: *Var*(*p*’) = *Var*[*E*(*p*’|*N*,*p*)]+*E*[*Var*(*p*’|*N*,*p*)].

Because *E*(*p*’|*N*,*p*) = *E*(*X*|*N*,*p*)/*N* and *Var*(*p*’|*N*,*p*) = *Var*(*X*|*N*,*p*)/*N* ^*2*^, and because *N* and *p* are independent, we then get: *Var*(*p*’) = *Var*(*p*)+*E*[*p*(1−*p*)]×*E*(1/*N*).

Using the properties $E(1/N)=\sum_{k=1}^{\infty }\left(\frac{1}{k}P(N=k)\right)$ and $\sum_{k=1}^{\infty }\frac{n_{vP}^{k}}{k!k}=\text{Ei}(n_{vP})-\text{ln}(n_{vP})-\gamma$,

where $\text{Ei}=\int_{-\infty }^{x}\frac{e^{t}}{t}dt$ is the exponential integral function and *γ* the Euler-Mascheroni constant, one can estimate the bottleneck size *n*_*vP*_ by numerically solving the following equation:

$$\frac{E(p)\left(1-E(p)\right)-Var(p)}{Var(p')-Var(p)}=\frac{e^{n_{vP}}-1}{\text{Ei}(n_{vP})-\text{ln}(n_{vP})-\gamma }.$$

We recommend using this estimation procedure when *N*_*v*_ values are below 20, i.e., when the relative estimation error is higher than 5%. The following lines of R code may be used to estimate *n*_*vP*_:

library(gsl)

EulerMaschCst<−(−digamma(1))

p<−c(…,.,…) # vector of initial frequencies

p_prime<−c(…,.,…) # vector of final frequencies

funestim<−function(n_vP) (mean(p)*(1−mean(p))−var(p))/(var(p_prime)−var(p)) −

(exp(n_vP)−1)/(expint_Ei(n_vP)−log(n_vP)−EulerMaschCst)

uniroot(funestim,interval = c(1e−6,600))
